# Low concentration trifluoperazine promotes proliferation and reduces calcium-dependent apoptosis in glioma cells

**DOI:** 10.1038/s41598-018-19413-y

**Published:** 2018-01-18

**Authors:** Yulin Wen, Yong Zhang, Jinbang Li, Feng Luo, Zhongxi Huang, Kunping Liu

**Affiliations:** 1 Department of Pathology, the Sixth Affiliated Hospital of Guangzhou Medical University, Qingyuan People’s Hospital, Qingyuan, 511518 China; 2 Department of Pathology, Qingyuan People’s Hospital, Jinan University, Qingyuan, 511518 China; 3 Division of Pathology, Guangdong Province Key Laboratory of Molecular Immunology and Antibody Engineering, Medical College, Jinan University, Guangzhou, 510632 China; 4 Guangdong Provincial Key Laboratory of Cancer Immunotherapy, Cancer Research Institute, School of Basic Medical Sciences, Southern Medical University, Guangzhou, 510515 China

## Abstract

Glioma patients constitute the greatest percentage of depressed neoplasm patients. These patients often require antidepressant treatment, but the effect of antidepressant drugs on glioma cells requires further evaluation. In the present study, we evaluated the effect of trifluoperazine (TFP) on the proliferation and apoptosis of glioma cells. Transcriptomic and bioinformatics analysis results suggested that antidepressant drugs, especially TFP, may upregulate the drug-resistant ability of glioma cells. A low concentration of TFP upregulated the viability of glioma cells. Colony formation and EdU assays confirmed that TFP treatment accelerates glioma cell proliferation, but no significant difference was found in the cell cycle distribution of glioma cells after treatment with TFP or control. Flow cytometry and TUNEL staining results suggested that TFP treatment decreased apoptosis in glioma cells. In addition, TFP treatment downregulated the intracellular Ca^2+^ concentration of glioma cells. *In vivo* experimental results indicated that TFP treatment promoted proliferation and reduced apoptosis in xenograft tumours in nude mice. Taken together, our results suggest that a low concentration of TFP promotes proliferation and reduces apoptosis in glioma cells both *in vitro* and *in vivo*. The potential harmful effects of antidepressant drugs on gliomas require further evaluation before their use in glioma patients.

## Introduction

Gliomas are the most common primary tumours of the central nervous system^1,2^. The overall prognosis for most glioma patients is poor, especially for glioblastoma patients^3^. The invasion of malignant glioma cells into the adjacent normal brain tissues often leads to incomplete surgical resection. Therefore, adjuvant treatments such as radiation and chemotherapy play important roles in the postoperative treatment of glioma patients. The chemotherapeutic drugs carmustine (BCNU) and temozolomide (TMZ) have been commonly used to treat gliomas for many years^4,5^. However, most gliomas eventually become drug resistant after chemotherapy^6–9^.

Recently, strenuous efforts have been made to re-evaluate conventional drugs to determine their potential effects on drug resistance^10^. For instance, the Connectivity Map database can be used to find connections among small molecules that share a mechanism of action, metabolites or physiological processes by providing a query interface to make inferences^11,12^.

In the present study, the glioma cell line SWOZ2 and the BCNU-resistant cell line SWOZ2-BCNU were subjected to transcriptomics analysis. The differentially expressed genes were submitted for Connectivity Map analysis to search for conventional drugs with the potential to impact the drug-resistant ability of SWOZ2 and SWOZ2-BCNU cells. One of the top-ranking candidates, trifluoperazine (TFP), was used to examine the effects on glioma cell proliferation and apoptosis.

## Results

### Data mining of gene expression profiles and drug screening in the Connectivity Map database

**Figure 1:**
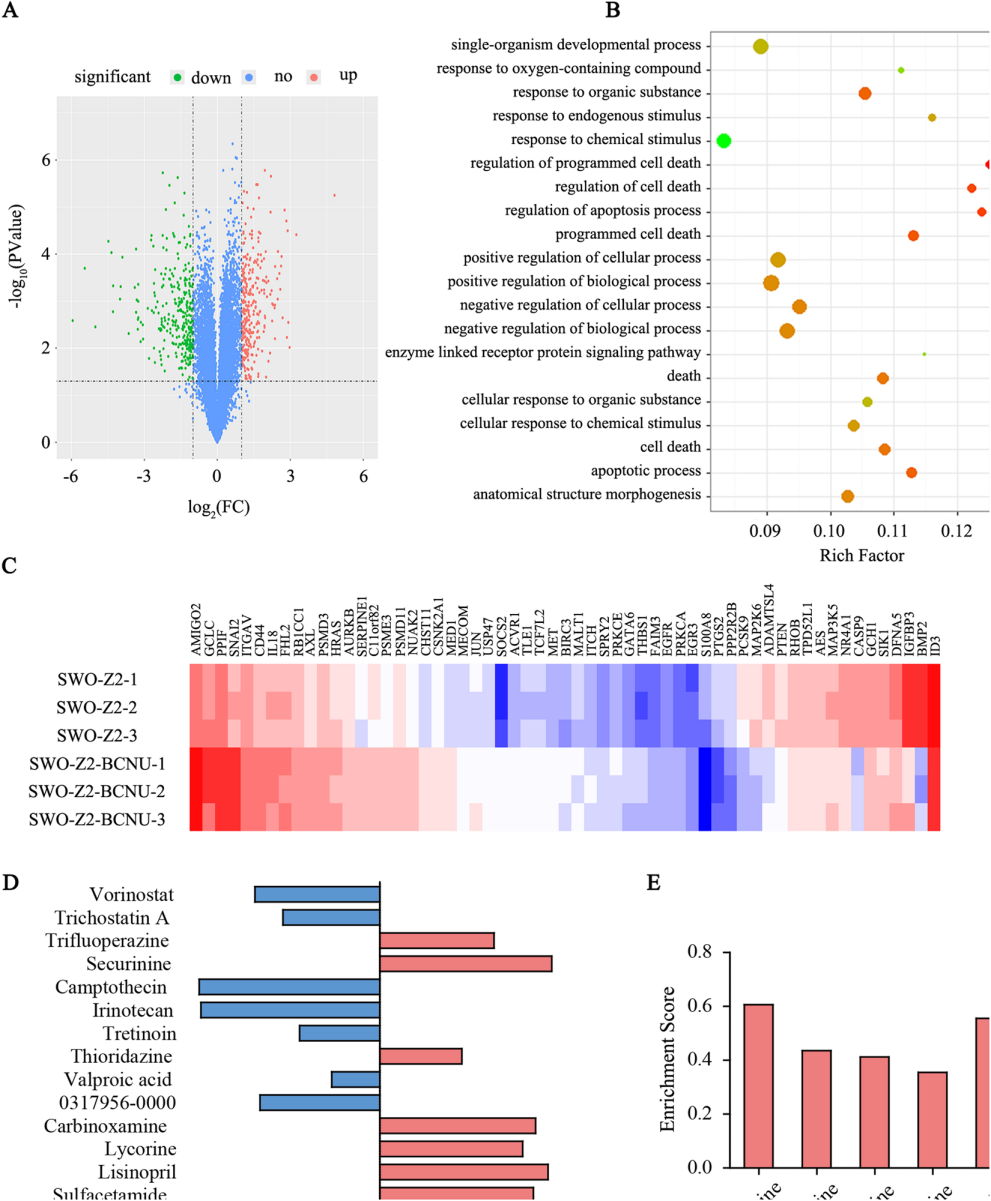
Data mining of gene expression profiles and drug screening in the Connectivity Map database. (**A**) Significance analysis of microarray (SAM) was performed to identify differentially expressed genes between SWOZ2 and SWOZ2-BCNU cells. (**B**) GO analysis of a cluster of differentially expressed genes between SWOZ2 and SWOZ2-BCNU cells was performed with GenCLiP software. (**C**) Supervised hierarchical clustering results showing 59 apoptosis-related differentially expressed genes between SWOZ2 and SWOZ2-BCNU. Samples were denoted in columns, and genes were denoted in rows. The mapped expression levels for all genes were depicted using a colour scale; highly expressed genes were indicated in red and poorly expressed genes in blue. (**D**) Gene expression signatures connect small molecules in the connectivity map database. Small molecules were picked after uploading the probe list of differentially expressed genes between SWOZ2 and SWOZ2-BCNU cells to the Connectivity Map database. The 15 highest-ranking small molecules correlated with SWOZ2 and SWOZ2-BCNU cell differentially expressed genes were listed on the left. Positive correlations were indicated in red and negative correlations in blue. (**E**) Enrichment scores of all phenothiazines among the 15 highest-ranking small molecules.

First, we extracted total RNA and conducted cDNA microarray analysis (Affymetrix Human Genome U133 Plus 2.0) for SWOZ2 and the matching BCNU-resistant cell line SWOZ2-BCNU. We screened differentially expressed genes by t-test on SWOZ2 and SWOZ2-BCNU gene chip data with three repeats. We then performed t-test analysis and identified 1,181 unique genes with aberrant expression in SWOZ2-BCNU when compared to SWOZ2 cells; there were 643 upregulated and 538 downregulated genes in SWOZ2-BCNU cells. The volcano plots of differentially expressed genes are shown in Fig. 1A. These differentially expressed genes were analysed by GenCliP 2.0 software to annotate gene functions. Sorting by the negative value of the log (p-value) of GO analysis is shown in Fig. 1B. The top-ranking terms were related to apoptosis and programmed cell death. These results suggest that the SWOZ2-BCNU cell line was likely to achieve BCNU resistance by escaping apoptosis. In Fig. 1C, supervised hierarchical clustering results showing 59 apoptosis-related differentially expressed genes between SWOZ2 and SWOZ2-BCNU.

To discover conventional drugs that target these gene sets, we uploaded the list of differentially expressed genes to the Connectivity Map database. Analysis results showed that among the top 20 conventional drugs, there were ten drugs with positive enrichment scores, while the other 10 were negative (Fig. 1D). Among these drugs, we found that TFP, thioridazine and fluphenazine had positive enrichment scores and that they all belong to a group of drugs called phenothiazines. We found that the enrichment scores of all phenothiazines were positive according to the Connectivity Map database analysis results (Fig. 1E). This suggests that phenothiazines might be able to promote drug resistance in glioma cells.

### Effect of TFP on cancer cell proliferation

**Figure 2:**
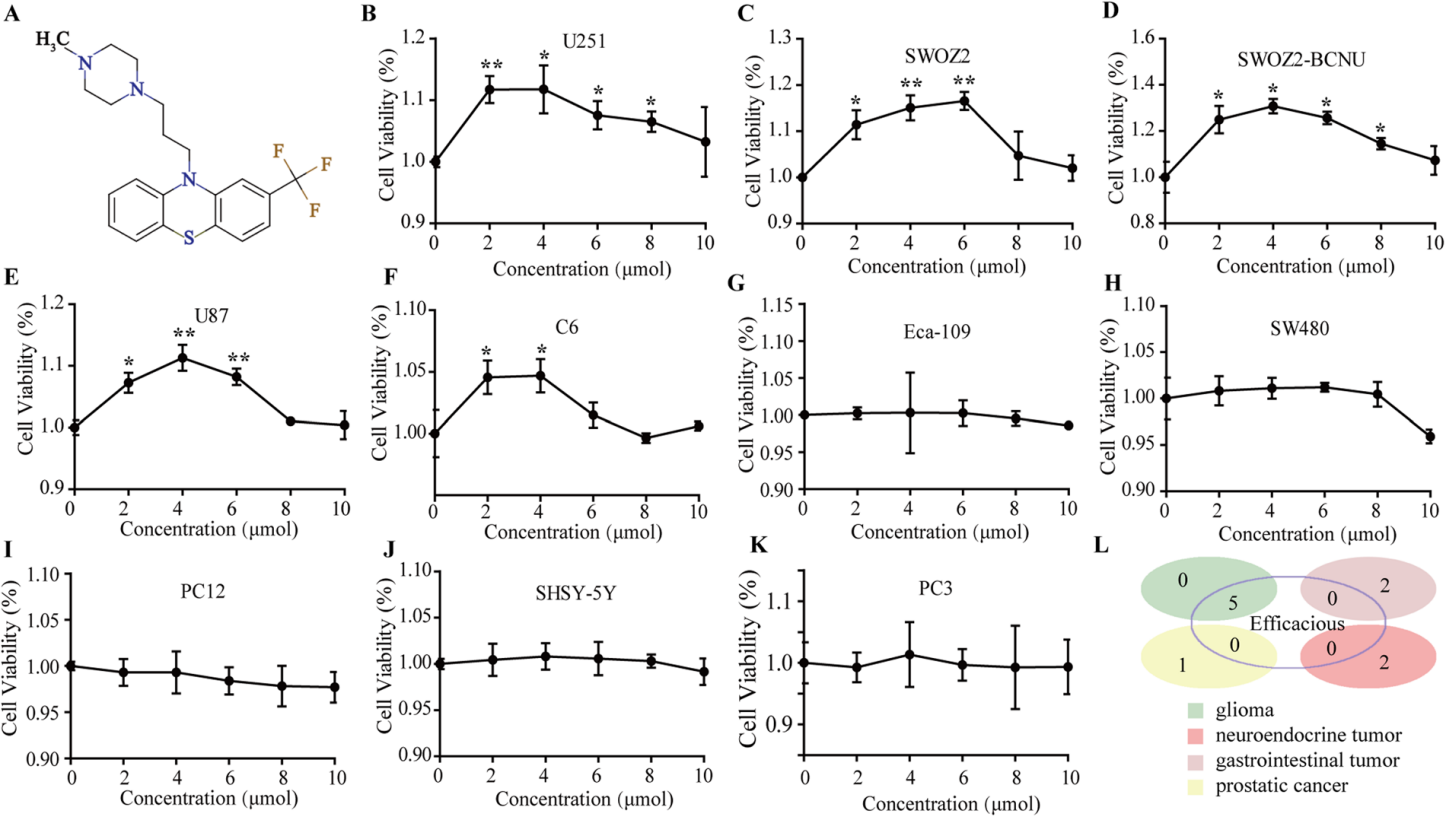
TFP accelerates *in vitro* cell proliferation of glioma-related cell types. (**A**) The molecular structure of TFP. (**B–F**) MTT assays for the glioma cells U87, U251, SWOZ2, SWOZ2-BCNU and C6. (**G**,**H**) MTT assays for the gastrointestinal tumour cells Eca-109 and SW480. (**I**,**J**) MTT assays for the neuroendocrine tumour cells PC12 and SHSY-5Y. (**K**) MTT assays for the prostatic cancer cell line PC3. (**L**) The proliferation-promoting effect of TFP on different cell types. Data are the mean ± SD deviation of triplicate determinations.

TFP is an anti-psychotic medicine that belongs to a group of drugs called phenothiazines. The molecular structure of TFP is shown in Fig. 2A. To determine the effect of TFP treatment on cell viability, different kinds of cell lines were treated with TFP (0 μM, 2 μM, 4 μM, 6 μM, 8 μM and 10 μM), and cell viability was measured 24 hours later. Interestingly, a low concentration of TFP (2 μM, 4 μM and 6 μM) treatment resulted in a statistically significant increase in the viability of glioma cell lines U87, U251, SWOZ2, SWOZ2-BCNU and C6 (Fig. 2B–F, p < 0.05). However, these effects were slight in the neuroendocrine cell lines PC12 and SHSY-5Y, the gastrointestinal cell lines Eca-109 and SW480, and the prostatic cell line PC3 (Fig. 2G–K, p > 0.05), suggesting that the proliferation promoting effect of TFP on cancer cells is highly selective.

### TFP treatment accelerates glioma cell proliferation *in vitro*

**Figure 3:**
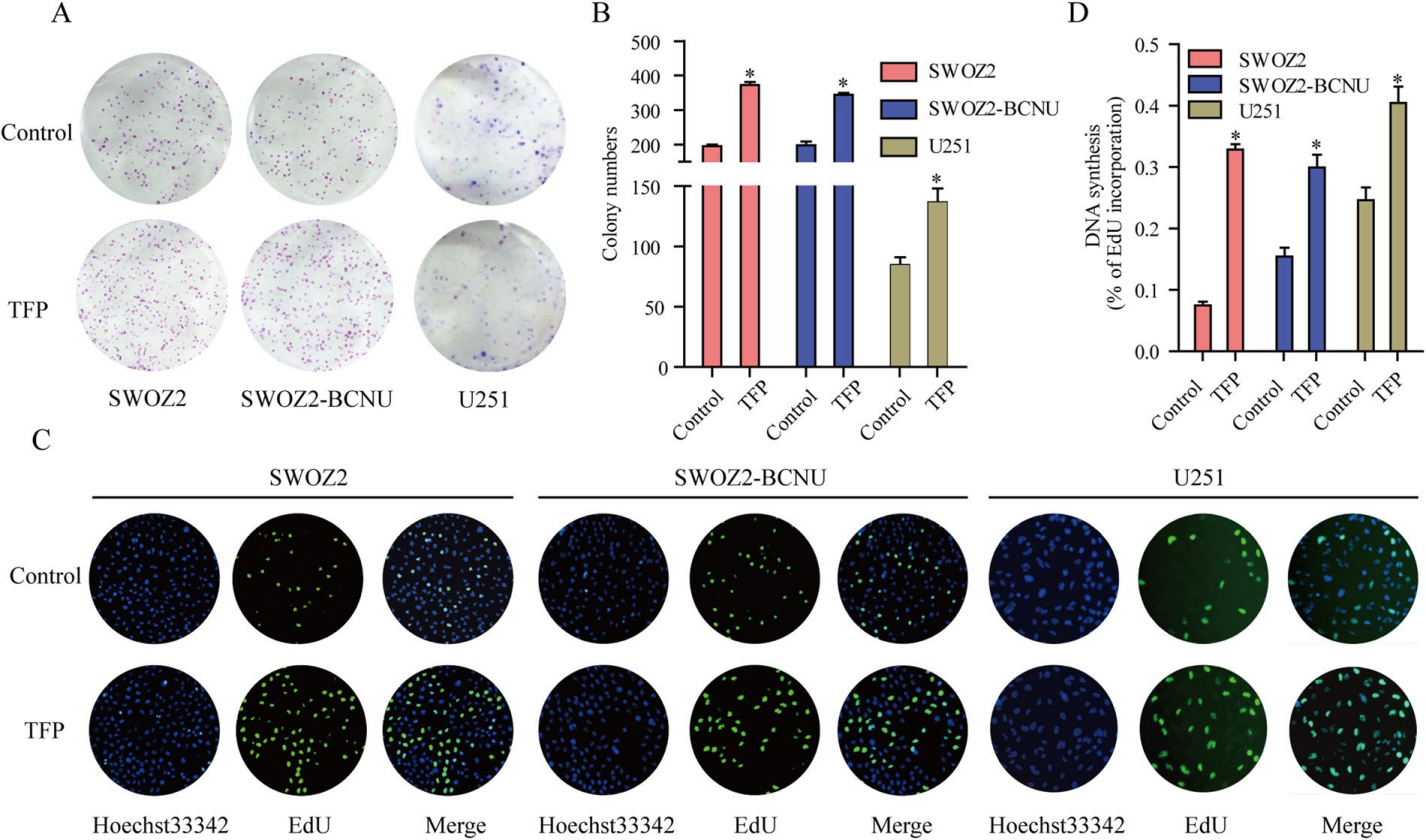
TFP accelerates glioma cell proliferation. (**A**,**B**) The colony forming ability of glioma cells SWOZ2, SWOZ2-BCNU and U251 was compared after treatment with TFP or control. Student’s t-test. Mean ± SEM, N = 3, **P < 0.01. (**C**,**D**) The cell proliferation status of glioma cells was also tested by EdU assay. Original magnification, ×100; scale bar 100 μm. Student’s t-test.

To further evaluate the effect of TFP on glioma cells, the colony forming ability of the glioma cells SWOZ2, SWOZ2-BCNU and U251 were examined after treatment with TFP (2 μM) or control. TFP treatment produced significantly more colonies compared with the control in SWOZ2, SWOZ2-BCNU and U251 cells (Fig. 3A and B, P < 0.01). After TFP treatment, the EdU assay was performed to determine the effect of TFP on glioma cell proliferation. TFP treatment significantly increased the percentage of EdU-positive cells compared with the control (Fig. 3C and D, P < 0.01).

### TFP treatment effect on the cell cycle distribution of glioma cells

**Figure 4:**
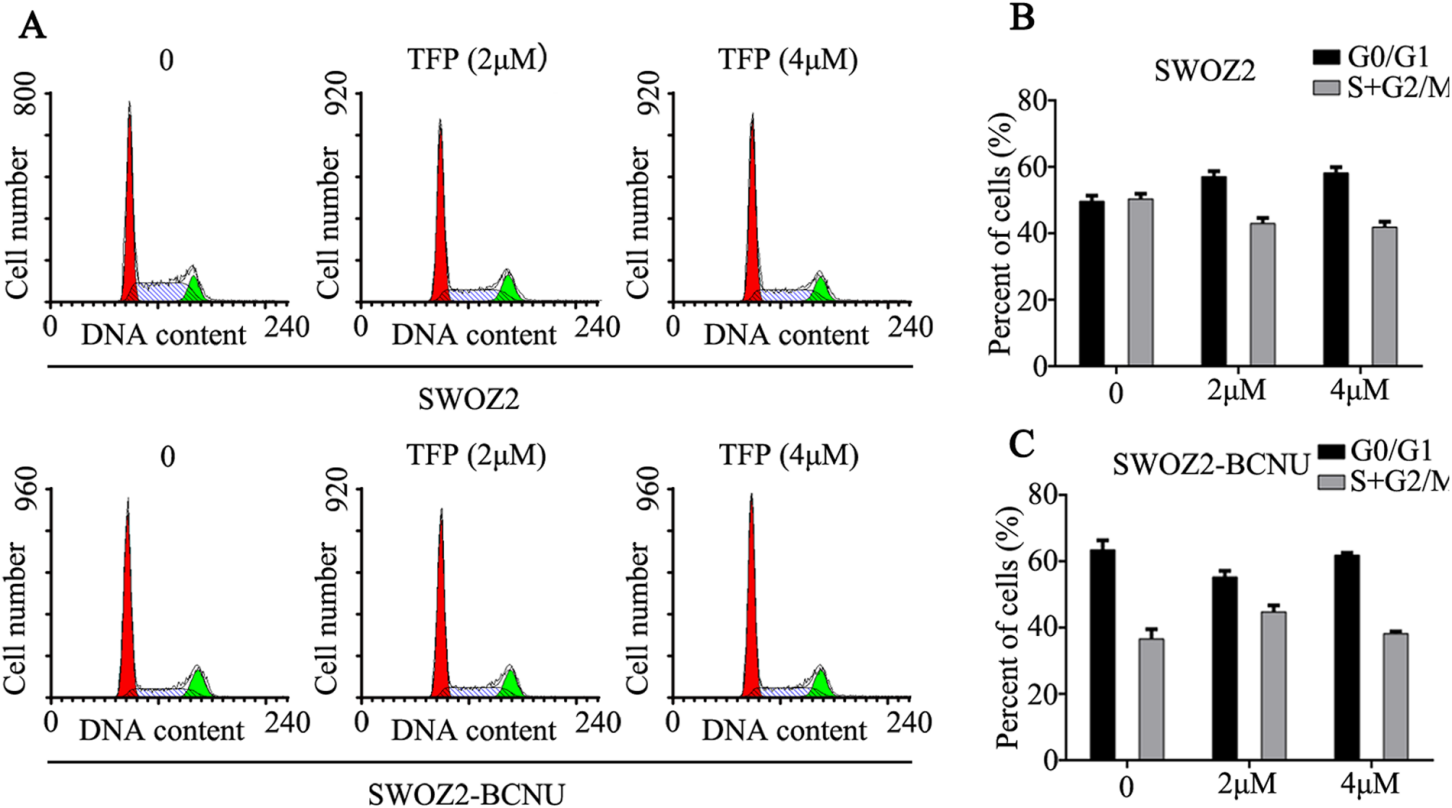
The effects of TFP on the cell cycle distribution of U251, SWOZ2 and SWOZ2-BCNU cells. (**A**) Flow cytometry analysis of the cell cycle distribution of SWOZ2, SWOZ2-BCNU and U251 cells after incubation with TFP or control. (**B–D**) The cell cycle distribution of SWOZ2, SWOZ2-BCNU and U251 cells after treatment with TFP or control. The results are presented as the mean ± SD of three experiments. *P < 0.05 compared with the control.

After incubation with TFP or control, SWOZ2, SWOZ2-BCNU and U251 cells were stained and cell cycle distribution was determined by flow cytometry (Fig. 4A). No significant difference was found in the cell cycle distribution of SWOZ2, SWOZ2-BCNU and U251 cells treated with or without TFP (Fig. 4B–D, P > 0.05).

### TFP treatment downregulated apoptosis of glioma cells

**Figure 5:**
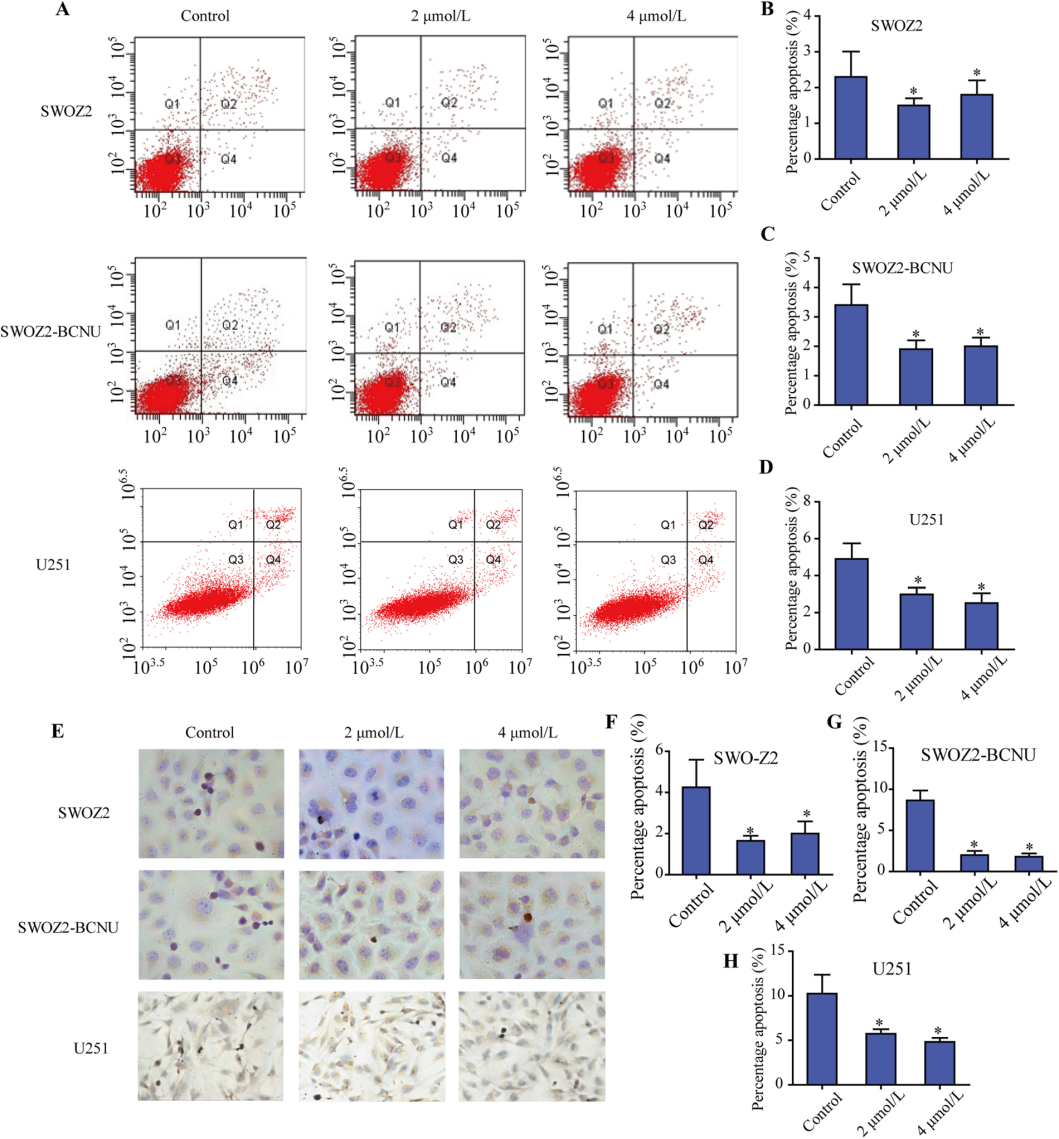
The effects of TFP on the apoptosis of SWOZ2, SWOZ2-BCNU and U251 cells. (**A**) Flow cytometry analysis of Annexin V-positive cells in SWOZ2, SWOZ2-BCNU and U251 cells incubated with TFP or control. (**B–D**) The rate of Annexin V-positive cells in SWOZ2, SWOZ2-BCNU and U251 cells after treatment. All Annexin V-positive cells were considered apoptotic cells, and their percentage was calculated among the total number of cells. (**E**) TUNEL staining assay on SWOZ2, SWOZ2-BCNU and U251 cells after TFP treatment (400×). (**F–H**) The rate of apoptotic cells in SWOZ2, SWOZ2-BCNU and U251 cells after treatment by TUNEL assay. Each bar represents the mean ± SD of three experiments. *P < 0.05 compared with the control.

After incubation with control or different concentrations of TFP (2 μM and 4 μM), the effect of TFP on the apoptosis of SWOZ2, SWOZ2-BCNU and U251 cells was examined using flow cytometry and TUNEL staining (Fig. 5A and E). We found that TFP treatment significantly decreased the apoptosis of glioma cells U251, SWOZ2 and SWOZ2-BCNU (Fig. 5B–D and F–H; P < 0.05). However, no significant difference was found in the apoptosis of glioma cells after treatment with different concentrations of TFP (2 μM vs. 4 μM, P > 0.05).

### TFP treatment promotes glioma growth *in vivo*

**Figure 6:**
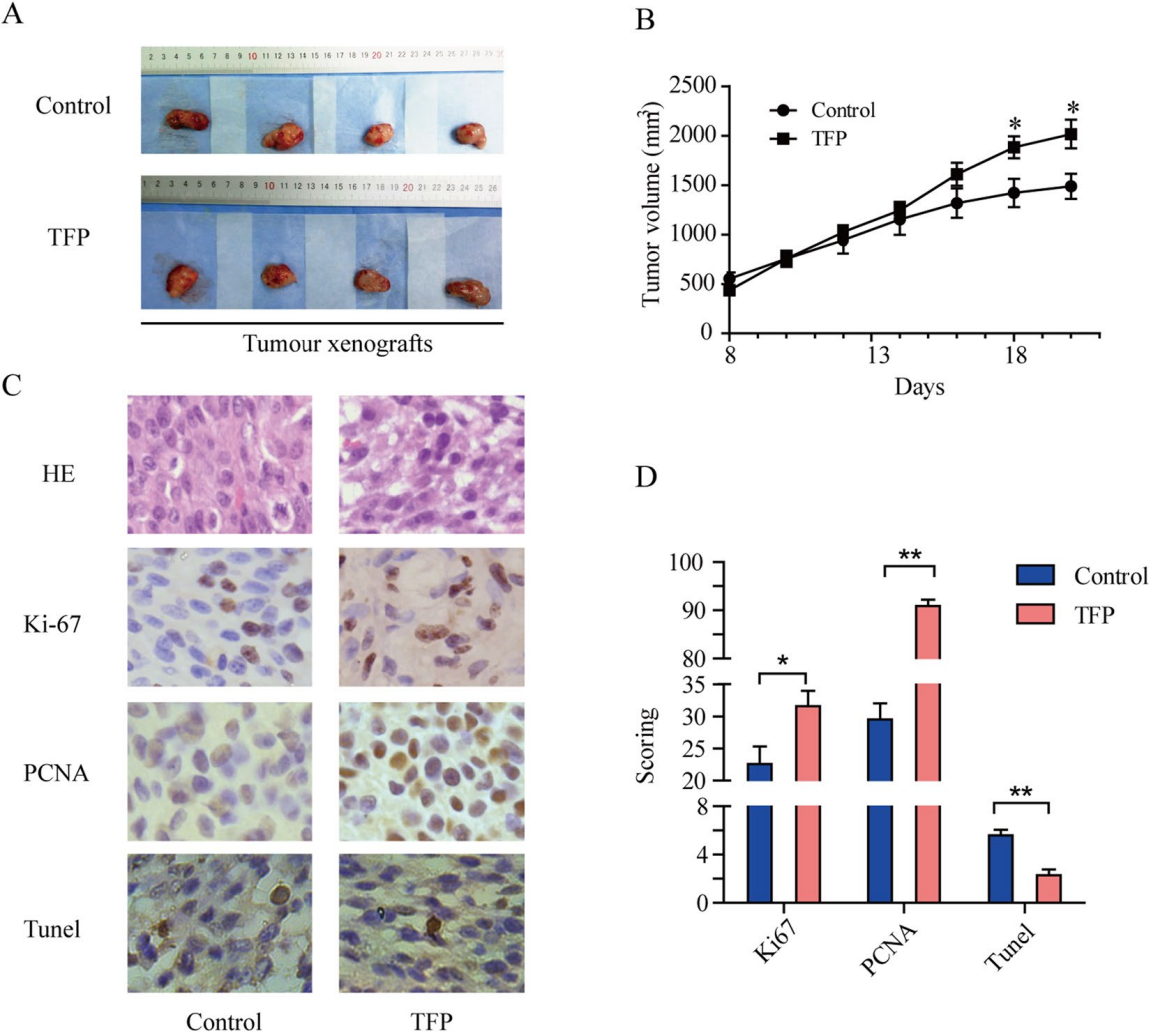
TFP promotes glioma cell proliferation *in vivo*. (**A**) Glioma cells were subcutaneously injected into nude mice (4 mice each group). The control group was injected with 0.9% sodium chloride and the treatment group was intraperitoneally injected with TFP (2 mg/kg, every 3 days) after the xenograft tumour formed. (**B**) Tumour sizes were measured every day using a Vernier calliper after the tumour became palpable. The tumour volumes were calculated as follows: volume = (D × d^2^)/2, where D indicated the longest diameter and d indicated the shortest diameter, plotted as the mean ± SEM. *P < 0.05. (**C**) Apoptosis, Ki67 and PCNA protein expression levels were examined using IHC in tumour tissues derived from the indicated mouse group (400×). (**D**) Immunohistochemical scoring and apoptosis scoring in indicated groups. Each bar represents the mean ± SD. *p < 0.05, **p < 0.01, ***p < 0.001.

To investigate whether TFP promotes tumour cell proliferation *in vivo*, glioma cell line U251 was injected subcutaneously into the dorsal flank of nude mice. After the xenograft tumour formed, TFP (2 mg/kg) or control (0.9% NaCl) was injected into nude mice every three days. Approximately 2~3 weeks later, the xenograft tumour volumes of the TFP-treated mice were significantly larger compared with controls (Fig. 6A and B, P < 0.05). We used an immunohistochemical assay to detect the expression levels of PCNA and Ki67 between these two groups. The immunohistochemical results showed that both the staining intensity and the number of hyperproliferative Ki-67 and PCNA positive tumour cells were significantly increased after TFP treatment (Fig. 6C and D, P < 0.05). In addition, TUNEL staining results suggested that TFP treatment significantly decreased the apoptosis of glioma cells in nude mice (Fig. 6C and D, P < 0.05).

### TFP treatment downregulated the intracellular Ca^2+^ concentration

**Figure 7:**
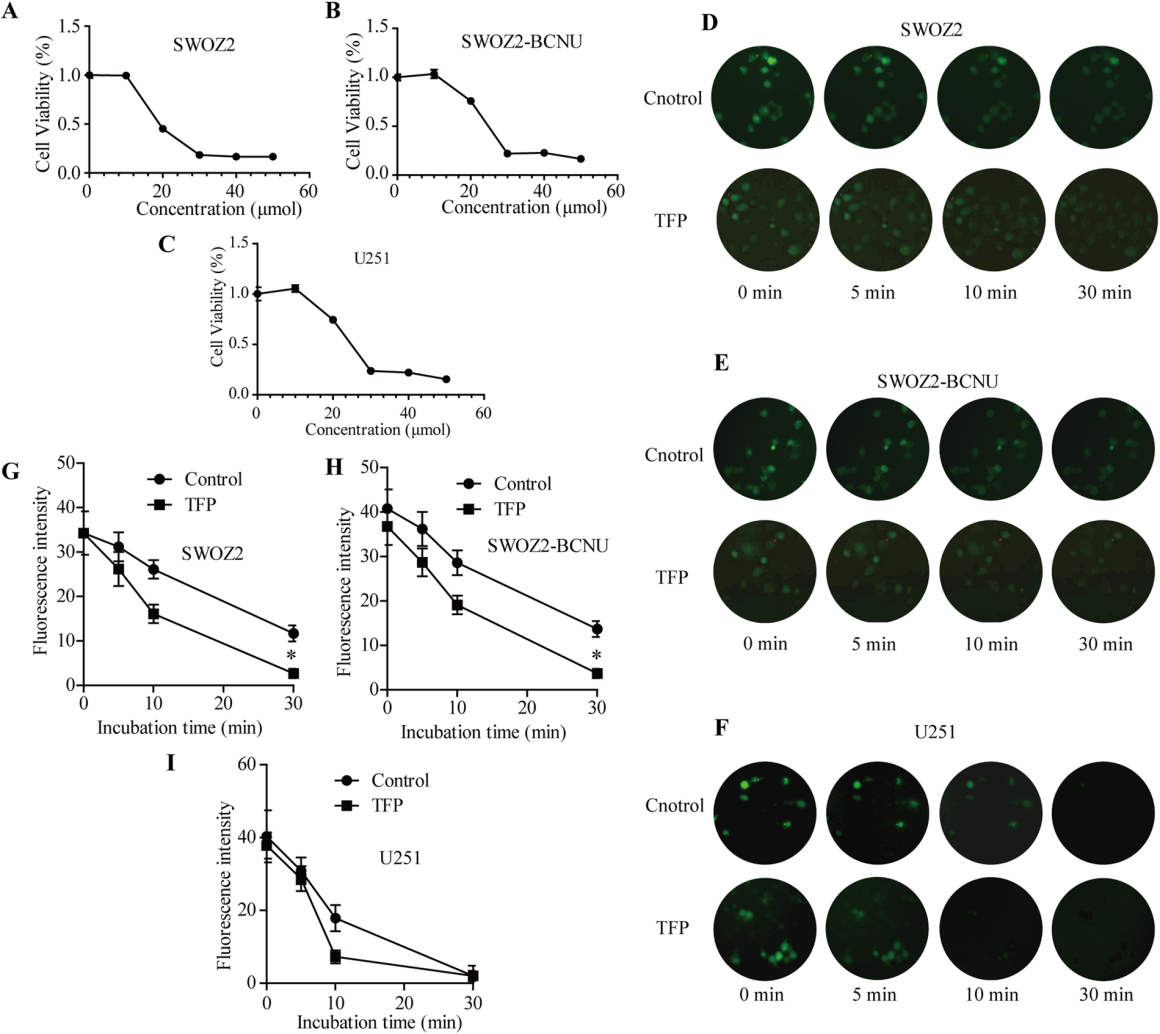
TFP treatment downregulated the intracellular Ca^2+^ concentration in glioma cells. (**A**–**C**) MTT assays for the glioma cells SWOZ2, SWOZ2-BCNU and U251 after TFP treatment. Cells were plated on 96-well plates and treated with different concentrations of TFP. The resulting number of viable cells was calculated by measuring the colour absorbance produced in each well. Data are the mean ± SD deviation of triplicate determinations. (**D**–**F**) Micrographic pictures of Fluo-3 fluorescence in SWOZ2, SWOZ2-BCNU and U251 cells treated with TFP at different time points. (**G**–**I**) The fluorescence intensity of Fluo-3-AM after TFP treatment in indicated cells at different time points. All values are presented as the mean ± SD of three experiments. *P < 0.05 compared with the control.

The MTT assay suggested that low concentrations of TFP treatment increased glioma cell viability (Fig. 2B–D, p < 0.05), but high concentrations of TFP treatment decreased SWOZ2, SWOZ2-BCNU and U251 cell viability (Fig. 7A–C, p < 0.05).

To confirm whether the TFP effect on glioma cell proliferation was associated with an altered intracellular Ca^2+^ concentration, the fluorescence intensity of Fluo-3-AM in SWOZ2, SWOZ2-BCNU and U251 cells was examined after treatment with TFP at different time points (5 min, 10 min and 30 min, Fig. 7D–F). Fluorescent Ca^2+^ imaging with Fluo-3-AM revealed that the Ca^2+^ concentration in glioma cells was reduced continuously after low-dose TFP (2 μM) treatment (Fig. 7G–I, p < 0.05).

## Discussion

Recently, efforts have been made to re-evaluate old drugs in the context of their potential helpful/harmful effects on cancer therapy. Currently, the CMap database has been successfully used to discover the molecular mechanisms underlying small molecule action and disease pathogenesis in various studies^13,14^. The CMap database is a collection of genome-wide transcriptional expression data from cultured human cells treated with different types of small molecules. As pattern-matching software, the CMap database uses the transitory feature of common gene-expression changes to connect small molecules, genes, and disease^15,16^. In the present study, the differentially expressed genes between the glioma cell line SWOZ2 and the BCNU-resistant cell line SWOZ2-BCNU were submitted to Connectivity Map analysis to search for conventional drugs with the potential to impact the drug-resistant ability of glioma cell lines. Interestingly, analysis results from the CMap database suggested that phenothiazines, especially TFP, can upregulate the drug-resistant ability of glioma cells. Because phenothiazines are widely used in glioma patients, these analysis results have important implications for glioma patient antidepressant treatment.

Brain neoplasm patients constitute the greatest percentage of depressed neoplasm patients. Recent research reported that approximately 90% of glioma patients were suffering from depression and neurobehavioural disorders^17,18^. These patients often require antidepressant treatment due to comorbid depression, but the effects of antidepressant drugs on glioma cell proliferation and apoptosis require further evaluation^19^. Recent studies indicated that both pro- and anticancer antidepressant drug properties were present in different studies. For instance, chlorimipramine, a tricyclic antidepressant drug, induced cytotoxic effects on glioma cells by increasing reactive oxygen species (ROS), inhibiting the complex III respiratory chain and causing caspase-activated apoptosis^20^. In addition, thioridazine exhibits anticancer action in cervical carcinoma, hepatoma, leukemia and breast cancer^21–24^. Previous study reported that high dose of trifluoperazine inhibit cancer stem cell growth and overcome drug resistance of lung cancer^16^. By contrast, clinically relevant doses of antidepressant drugs stimulated malignant growth in rodents^25^, consistent with our experimental results.

In the present study, we first investigated the effect of TFP on the viability of cancer cells. Interestingly, our data showed that low concentrations of TFP resulted in significantly increased viability in glioma cell types, but these effects were slight in neuroendocrine, gastrointestinal and prostatic cancer cell lines. These experimental results suggest that the proliferative effect of TFP on cancer cells is highly selective. Flow cytometry results indicated that apoptosis was decreased in TFP-treated glioma cells. The colony formation and EdU assays also demonstrated that the proliferative ability of glioma cells was increased after TFP treatment. The stronger staining intensity and the increased number of hyperproliferative Ki-67 and PCNA positive tumour cells in TFP-treated nude mice suggested that TFP also promotes glioma cell growth *in vivo*. In agreement with the results of Wen *et al*.^25^, the present study also suggested that clinically relevant doses of TFP promote proliferation and reduce apoptosis in cancer cells. By contrast, Yeh and colleagues reported that TFP inhibits cancer stem cell growth and overcomes drug resistance in lung cancer^26^. In addition, Kang *et al*. reported that a high concentration of TFP inhibits glioblastoma invasion by binding to calmodulin and disinhibiting the calcium release channel IP3R^27^. Therefore, the role of TFP on the biological properties of cancer cells may vary between cell types and drug concentrations.

Ca^2+^-mediated cell connectivity and plasticity have been characterized as important mechanisms and unique features of the central nervous system^28^. In addition, Ca^2+^-induced apoptosis is an important component of programmed cell death^29^. A decreased intracellular Ca^2+^ concentration could reduce calcium-dependent apoptosis^30^. Interestingly, studies have indicated that the antidepressant drugs fluoxetine and TFP altered the intracellular Ca^2+^ concentration. For instance, Liu *et al*. reported that a low concentration of TFP (1 μM) treatment could inhibit the calcium overload process and ameliorate H_2_O_2_-induced oxidative stress and apoptosis^31^. Our results show that a low concentration of TFP reduced the intracellular Ca^2+^ concentration, indicating that TFP may reduce calcium-dependent apoptosis in glioma cells. By contrast, Kang *et al*. indicated that a high concentration of TFP could increase the intracellular Ca^2+^ concentration in glioma cells. Taken together, different concentrations of TFP may have different effects on the Ca^2+^ distribution.

Briefly, our *in vitro* and *in vivo* experimental results suggested that low concentrations of TFP promote glioma cell proliferation. TFP may reduce the calcium-dependent apoptosis of glioma cells. However, the underlying molecular mechanism of low concentration TFP in the regulation of glioma cell proliferation and apoptosis was not fully evaluated in the present study. The potential harmful effect of antidepressant drugs on glioma cells, especially TFP, requires further evaluation before using these drugs in glioma patients with depression and neurobehavioural disorders.

## Materials and Methods

### Cell culture

Cell lines U87, U251, SH-SY5Y, C6 and PC-12 were obtained from the Shanghai Institutes for Biological Sciences (SIBS, Shanghai, China). The glioma cell line SWO was established in 1985 from a 12 years old male patient by our laboratory at the Department of Pathology, Medical School, Ji Nan University^32^. The human glioma cell lines SWOZ2 and BCNU-resistant SWOZ2-BCNU were the sublines of SWO-38 cells^33,34^. The supplementary material describes in detail the origin, morphology and biological behaviour of the SWO cell line. U87, U251 and SH-SY5Y cell lines were cultured in DMEM (HyClone), while SWOZ2, SWOZ2-BCNU, C6, PC12, PC3, SW-480 and Eca-109 cell lines were maintained in RPMI-1640 medium (HyClone). All of these cell lines were supplemented in medium with 10% foetal bovine serum (Every Green, China) and 100 U/ml penicillin/streptomycin at 37 °C in a humidified 5% CO_2_ incubator.

### Microarray hybridization and analysis

The SWOZ2 and SWOZ2-BCNU cell lines were analysed using Affymetrix Human Genome U133 Plus 2.0 microarrays with three biologic repeats. Briefly, the total RNA was isolated using Trizol reagent (Invitrogen, Carlsbad, CA) according to the manufacturer’s instructions. RNA samples were labelled and hybridized as suggested by the standard protocol. Microarray scanning and data acquisition were performed at CapitalBio Genomics Co. (Dongguan, China) using Affymetrix (Santa Clara, CA) recommended equipment and procedures. The data were analysed with Microarray Suite version 5.0 (MAS 5.0).

### Screening differentially expressed genes, functional analysis and Connectivity Map analysis

To identify genes that were differentially expressed between SWOZ2 and SWOZ2-BCNU cell lines, a screening filter consisting of the following criteria was applied: (1) probe sets that could be correctly mapped to human genes and had a link in the Ensembl database (http://www.ensembl.org/); (2) a fold change larger than two in each repeat; (3) at least one expression value larger than 50 in each repeat; and (4) detection P < 0.05 in each repeat. GenCliP 2.0 was used to analyse the major biologic processes and molecular functions of these differentially expressed genes. The 1,181 differentially expressed genes between the SWOZ2 and BCNU-resistant SWOZ2-BCNU cell lines were submitted to the Connectivity Map online web tool (http://www.broadinstitute.org/cmap/).

### Cell viability assay

Cells were plated onto 96-well plates in triplicate at a density of 4 × 10^3^ cells per well and allowed to adhere overnight in 1640 or DMEM medium. Cells were incubated with TFP (Sigma-Aldrich Bio, USA) at various concentrations for 24 hr. After incubation, 10 μM/well of MTT solution (5 mg/ml phosphate buffered saline) was added and incubated for 4 hours. The medium was aspirated and replaced with 100 μl/well of dimethyl sulphoxide to dissolve the formazan salt that formed. The colour intensity of the formazan solution was measured at 570 nm using a microplate spectrophotometer (ELx800 Biotech Instruments, USA).

### EdU assay

The Cell-Light TM EdU staining kit (Ribobio, GuangZhou, China) was used for the *in vitro* labelling of the nucleus of dividing cells. The glioma cells were treated with 2 μM TFP and then with 10 μM EdU. After 16 hours, the cells were fixed. Then, the cells were examined on a laser scanning confocal microscope (Zeiss).

### Flow cytometric analysis

Cells were seeded in six-well plates, treated with Tet at IC20 for 24 hours and then exposed to radiation. Before the cells were analysed, 100 ml of binding buffer containing 1 ml of 100 mg/ml propidium iodide (PI) was added to these cells, and the cells were incubated for 30 min in the dark. Analyses were performed using a FACScan flow cytometer (Beckman Coulter, USA). Cell cycle distribution was calculated based on DNA plots using MultiCycle software (Phoenix Flow Systems, San Diego, CA, USA).

### Colony formation assay

For every cell line, 200 cells were seeded in 6-well plates and cultured in 1640 or DMEM with or without 2 μM TFP. Ten days after seeding, cells were fixed with 4% paraformaldehyde for 30 min at room temperature. Colonies were stained with crystal violet for 10 min (Beyotime Biotechnology, China) and then visualized using a digital camera (Sony Corporation, Japan).

### Animals and tumour xenografts

Animal experimentation was approved by the Ethics Committee of Animal Research at Jinan University, Guangzhou, China. All experimental protocols were performed in accordance with the associated national guidelines from the Ministry of Science and Technology of China. Male athymic balb/c mice at 5–6 weeks old were obtained from the Medical Science Experimentation Center of Sun yat-sen University (Guangzhou, China) for tumour inoculation. Briefly, U251 cells (3 × 10^6^ in 100 μL Matrigel) were inoculated subcutaneously into the right flanks of mice. Three days after tumour inoculation, mice were divided into two groups (4 mice/group): the control group injected with 0.9% sodium chloride and the treatment group intraperitoneally injected with TFP (2 mg/kg, every 3 days) after xenograft tumour formation. Tumour sizes were measured every day using a Vernier calliper after the tumour became palpable. The tumour volumes were calculated as follows: volume = (D × d^2^)/2, where D indicated the longest diameter and d indicated the shortest diameter. At the end of the experiment, tumours were removed from mice, fixed with formalin and embedded routinely with paraffin for immunohistochemistry staining.

### Immunohistochemistry

For immunohistochemical staining (IHC), the anti-Ki67 (CST) dilution was 1:500. The anti-PCNA (CST) dilution was 1:500. The slides were incubated with primary antibody overnight at 4 °C. For negative controls, the primary antibody was replaced by normal mouse serum. Horseradish peroxidase (HRP)-labelled secondary antibody from the MaxVisionTM HRP-Polymer anti-mouse immunohistochemistry (IHC) kit was applied, and the slides were incubated for 30 min at room temperature, followed by a 5 min incubation at room temperature with DAB. Finally, the sections were counterstained with haematoxylin and mounted with Permount (BIOS, Beijing, China). The results were visualized and photographed under a light microscope.

### Intracellular calcium ([Ca^2+^]_i_) measurement

To measure alterations in the intracellular Ca^2+^ concentration after TFP treatment, glioma cells were suspended and incubated with 5 μM Fluo-3-AM Molecular Probes (Beyotime Biotechnology) for 45 min at 37 °C. The fluorescence intensities of Fluo-3-AM in U251, SWOZ2 and SWOZ2-BCNU cells were visualized using a fluorescence microscope (Leica) after treatment with TFP (2 μM) at different time points (5 min, 10 min and 30 min).

## Electronic supplementary material

Supplementary material

## References

[1] Dolecek TA, Propp JM, Stroup NE, Kruchko C (2012). CBTRUS statistical report: primary brain and central nervous system tumors diagnosed in the United States in 2005-2009. Neuro Oncol.

[2] Torre LA (2015). Global cancer statistics, 2012. CA Cancer J Clin.

[3] Lacroix M (2001). A multivariate analysis of 416 patients with glioblastoma multiforme: prognosis, extent of resection, and survival. J Neurosurg.

[4] Sai K, Yang QY, Shen D, Chen ZP (2013). Chemotherapy for gliomas in mainland China: An overview. Oncol Lett.

[5] Sarkaria JN (2008). Mechanisms of chemoresistance to alkylating agents in malignant glioma. Clin Cancer Res.

[6] Ho VK (2014). Changing incidence and improved survival of gliomas. Eur J Cancer.

[7] Kang MK, Kang SK (2007). Tumorigenesis of chemotherapeutic drug-resistant cancer stem-like cells in brain glioma. Stem Cells Dev.

[8] Johnson BE (2014). Mutational analysis reveals the origin and therapy-driven evolution of recurrent glioma. Science.

[9] Hirst TC (2013). Systematic review and meta-analysis of temozolomide in animal models of glioma: was clinical efficacy predicted?. Br J Cancer.

[10] Gatti L, Cassinelli G, Zaffaroni N, Lanzi C, Perego P (2015). New mechanisms for old drugs: Insights into DNA-unrelated effects of platinum compounds and drug resistance determinants. Drug Resist Updat.

[11] Lamb J (2006). The Connectivity Map: using gene-expression signatures to connect small molecules, genes, and disease. Science.

[12] Lamb J (2007). The Connectivity Map: a new tool for biomedical research. Nat Rev Cancer.

[13] Yu XM (2006). The Role of Intracellular Sodium in the Regulation of NMDA-Receptor-Mediated Channel Activity and Toxicity. Mol Neurobiol.

[14] Wang HG (1999). Ca2+-induced apoptosis through calcineurin dephosphorylation of BAD. Science.

[15] Zhang XZ (2012). Analyzing gene expression profile in K562 cells exposed to sodium valproate using microarray combined with the connectivity map database. J Biomed Biotechnol.

[16] Yeh CT (2012). Trifluoperazine, an antipsychotic agent, inhibits cancer stem cell growth and overcomes drug resistance of lung cancer. Am J Respir Crit Care Med.

[17] Bielecka, A. M. & Obuchowicz, E. Antidepressant drugs can modify cytotoxic action of temozolomide. *Eur J Cancer Care (Engl)* (2016). 10.1111/ecc.12551 27480195

[18] Brandes LJ (1992). Stimulation of malignant growth in rodents by antidepressant drugs at clinically relevant doses. Cancer Res.

[19] Xu Y (2016). MicroRNA-122 confers sorafenib resistance to hepatocellular carcinoma cells by targeting IGF-1R to regulate RAS/RAF/ERK signaling pathways. Cancer Lett.

[20] Martins-Neves SR (2016). Chemotherapy induces stemness in osteosarcoma cells through activation of Wnt/beta-catenin signaling. Cancer Lett.

[21] Mao M, Yu T, Hu J, Hu L (2015). Dopamine D2 receptor blocker thioridazine induces cell death in human uterine cervical carcinoma cell line SiHa. J Obstet Gynaecol Res.

[22] Yin T (2015). Dopamine receptor antagonist thioridazine inhibits tumor growth in a murine breast cancer model. Mol Med Rep.

[23] Sachlos E (2012). Identification of drugs including a dopamine receptor antagonist that selectively target cancer stem cells. CELL.

[24] Lu M (2015). Roles of dopamine receptors and their antagonist thioridazine in hepatoma metastasis. Onco Targets Ther.

[25] Wen Q (2015). Connectivity mapping using a combined gene signature from multiple colorectal cancer datasets identified candidate drugs including existing chemotherapies. BMC Syst Biol.

[26] Chien JM (2011). The mechanism of sertraline-induced [Ca2+]i rise in human OC2 oral cancer cells. Hum Exp Toxicol.

[27] Kang S (2017). Trifluoperazine, a Well-Known Antipsychotic, Inhibits Glioblastoma Invasion by Binding to Calmodulin and Disinhibiting Calcium Release Channel IP3R. Mol Cancer Ther.

[28] Liu S, Han Y, Zhang T, Yang Z (2011). Protective effect of trifluoperazine on hydrogen peroxide-induced apoptosis in PC12 cells. Brain Res Bull.

[29] Yuan K (2015). Calmodulin antagonists promote TRA-8 therapy of resistant pancreatic cancer. Oncotarget.

[30] Bowie M (2015). Fluoxetine induces cytotoxic endoplasmic reticulum stress and autophagy in triple negative breast cancer. World J Clin Oncol.

[31] Souza DSP, Saraiva DF, Ferraz DCD, Scofano HM, de Carvalho-Alves PC (2007). Trifluoperazine protects brain plasma membrane Ca(2+)-ATPase from oxidative damaging. Exp Brain Res.

[32] Situ R (1987). Establishement of human brain malignant glioma cell line (SWO-38) and observation of its biologic properties. Chin J Cancer.

[33] Zhong XY, Chen YX, Ye SF (2000). Establishment of two cell sublines from SWO-38 glioma cells: an immunohisto-chemical and ultrastructural Study. Int J Modern Cancer Therapy.

[34] Lin C, Liang Y, Zhu H, Zhang J, Zhong X (2012). R280T mutation of p53 gene promotes proliferation of human glioma cells through GSK-3beta/PTEN pathway. Neurosci Lett.

