# Supplementary material for: Low concentration trifluoperazine promotes proliferation and reduces calcium-dependent apoptosis in glioma cells
Source: Sci Rep. 2018 Jan 18;8:1147. doi: 10.1038/s41598-018-19413-y (PMC5773581; doi:10.1038/s41598-018-19413-y)
Supplement: Supplementary file 1 — Supplementary material [file 41598_2018_19413_MOESM1_ESM.pdf]

Supplementary Information

- Title of manuscript:

Low concentration trifluoperazine promotes proliferation and reduces calcium-dependent apoptosis in glioma cells

- Authors:

Yulin Wen[a,b,*], Yong Zhang[c,*], Jinbang Li[a,b], Feng Luo[a,b], Zhongxi Huang [d], Kunping Liu[a,b]

Following are the main points supported that the SWO cell lines used as models for glioblastoma in present study can be representative of glioblastoma patho-physiology.

Firstly, clear and definite origin of SWO cell lines. Glioma cell line SWO was established in 1985, 32 years ago, from a 12 years old male patient by our laboratory, Department of Pathology, Medical School, Ji Nan University[1]. The human glioma cell lines SWOZ2 and BCNU-resistant SWOZ2-BCNU were the sublines of SWO cells[2]. The postoperative specimen for this patient was pathological diagnosed with fibrillar astrocytoma (Fig. S1A, Degree I). Tumor specimen taken from nude mice after transplanted with SWO cells for three weeks was pathological diagnosed with malignant astrocytoma.

Secondly, in vivo experimental results revealed the malignancy of glioblastoma cell line SWO. SWO cells were injected subcutaneously into 5 nude mice. At about 3 weeks after implantation, the rate of tumor formation was 100% (Fig. S1B). Tumor specimen taken from nude mice PTAH staining showed positive glial fibers and was pathological diagnosed with malignant astrocytoma, coincidence with origin patient's pathologic diagnosis. Local lymph node metastasis rate was 80% after tumor formation for 15 days[1].

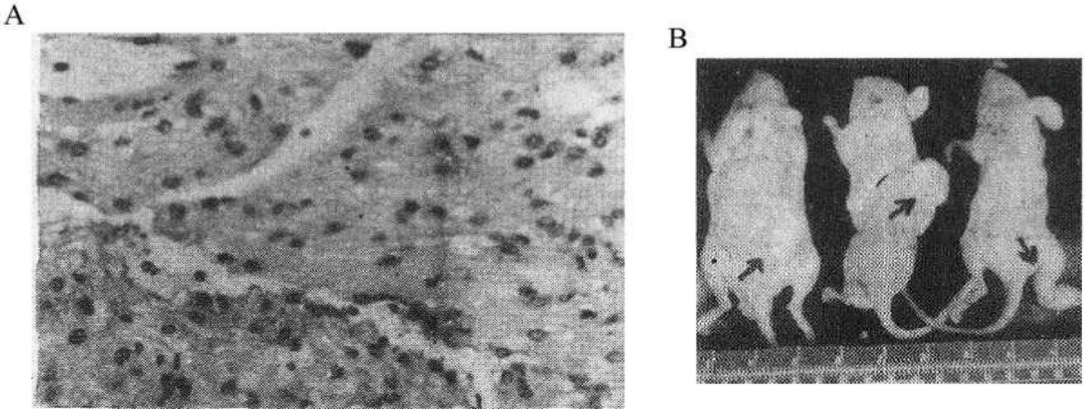

Fig. S1. Origin and in vivo malignancy of glioblastoma cell line SWO. A, HE stain (**200**×) were performed to observe the histomorphology of postoperative specimen for the 12 years old male patient. B, $1.0\text{-}1.2\times10^{6}$ SWO cells were injected subcutaneously into nude mice. At about 3 weeks after implantation, SWO cells produced tumors in nude mice.

Thirdly, in vitro experimental results revealed the malignancies of glioblastoma cell line SWO. SWO cell line has been transfered for 62 passages in a period of 8 months after separated from patient. Cell morphology and biological behavior were observed during this period.

Light microscope: there were two kinds of cellular morphology at the early of glioblastoma

cells separated from patient. For one was glial cell, typical star like, cytoplasmic projections with slender branching. The other was epithelioid glial cells, with polygonal epithelioid, abundant cytoplasm, no obvious polarity, in a single epithelioid cell background focal accumulation of cells showed irregular growth pattern. After 3-4 generation, the number of star like glial cells gradually decreased or disappeared, and the pure epithelioid glial cells constitute the SWO cell line (Fig. S2A) [(1)].

Transmission electron microscope: the second generations of SWO cells were suspension and centrifugation. After fixation, embedding, sectioning, the tumor cells were epithelioid like, slightly different size, and cell membrane surface has many fine finger prominent under the electron microscope. Free ribosomes were visible in the cytoplasm, with lots of poly ribosome among them. Moderate amount of endoplasmic reticulum were saccular dilatation partly. The nuclei of tumor cells were different in size. Dual nuclei, giant nuclei and abnormal nuclei were found in the nuclei, with obvious nuclear atypia. The nuclear have abundant chromatin; mostly heterochromatin were massive and distributed on the edge of nuclear envelope, abound with nuclear pore[(1)].

Scanning electron microscope: the scanning electron microscope was used to obvious SWO cells after separated from patient and monolayer cultured in square cover slips (Fig. S2C). The cell surface was mulberry like, with small vesicles, eeiticbleb and microvilli. Most tumor cells extended filar pseudopodia around and adhered at the slides, the diameter of distinct length pseudopod were about 0.2-8μm. Some bifurcate were found at the end of the pseudopodia, dilated vesicles were visible sometimes. A few tumor cells extended thick pseudopods[(1)].

Chromosome analysis of SWO cells: Whole chromosome of SWO cells were observed after colchicine treatment and Giemsa staining (Fig. S2E). The chromosome numbers of 50 metaphase SWO cells were in the range of 80-115, between sub-tetraploid to hyper-tetraploid. Apart from the abnormal increase in the number of chromosomes, aberrations chromosome, large chromosomes, double point, all ring, semicircular and other abnormal chromosome structures of malignant tumor cells were also found in SWO cells[(1)].

Cell division index and growth curve: HE stain (200×) was performed to observe the 22 generation of SWO cells. Daily counts of mitotic figures in 2,00 SWO cells (Fig. S2D) and cell division index were calculated. Results show that after cultured for 3-5 days cell division reached the peak, the cell division index was 54-56.5% (Fig. S2F). Cell growth curve of SWO cells was

also calculated, the doubling time of SWO cells was about 46.6 hours. The proliferation of SWO cells was slower in the first day, and then rose to the highest level in the fifth day, and the maximum growth rate of 1.7×10^5 cells (per dish) was about 5.2 times (Fig. S2G)[1].

Same reaction compared with classic glioma cells after trifluoperazine (TFP) treatment. In present study, low concentrations of TFP (2μM, 4 μM and 6 μM) exposure resulted in a significant increase in the viability of classic gliomas cell types U87, U251 and C6 (Fig. S2B-F, $p < 0.05$), as well as our cell lines SWOZ2 and SWOZ2-BCNU. However, these effects were slight in neuroendocrine tumor cells, gastrointestinal tumor cells and prostatic cancer cells.

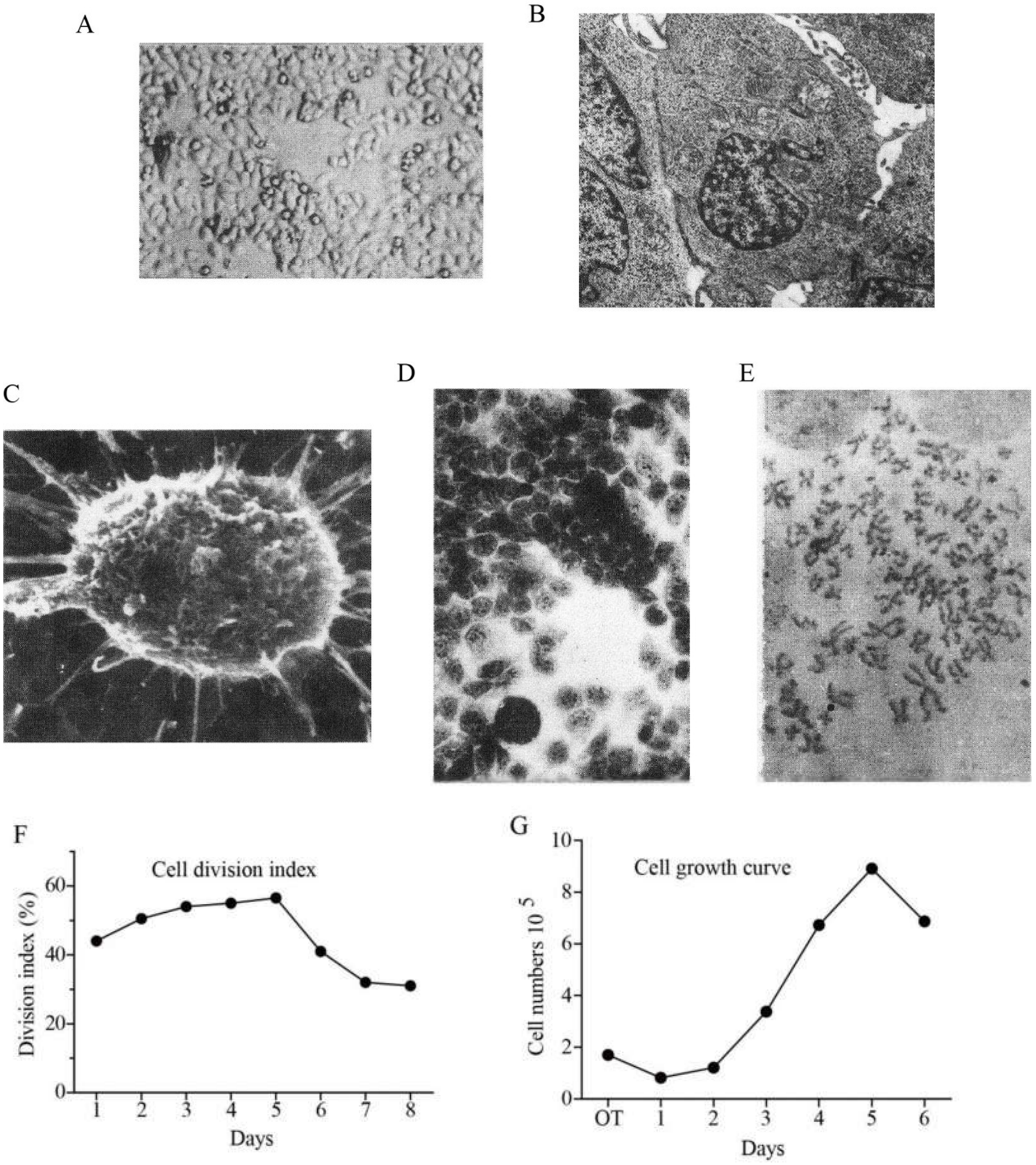

Fig. S2. In vitro malignancies of glioblastoma cell line SWO. A, the morphology of SWO cells after separated from patient and monolayer cultured for 4 days. Phase contrast microscope, 300×.

B, the ultrastructure of SWO cells after separated from patient and monolayer cultured for 4 days. Electron microscope, 5,300×. C, the scanning electron microscope picture of SWO cells after separated from patient and monolayer cultured for 4 days. Scanning electron microscope, 4,100×. D, the epithelioid cell morphology of SWO cells after separated from patient and monolayer cultured for 4 days, several pathologic mitotic cells were found. HE staining, 400×. E, whole chromosome of SWO cells, the chromosome number of SWO cells was up to sub-tetraploid. Giemsa staining, 1000×. F, cell division index of SWO cells after separated from patient and cultured for 8 days. G, cell growth curve of SWO cells after separated from patient and cultured for 6 days.

Finally, the malignancies of glioblastoma cell line SWO have been employed extensively by various studies in the past 32 years, provided valuable knowledge about this cell line[(3-16)]. The in vivo and in vitro experimental results from these studies also suggested that SWO cell lines can be representative of glioblastoma patho-physiology partly.

Reference:

(1) Situ R, Wang H H, Wang J H, et al. Establishement of human brain malignant glioma cell line (SWO-38) and observation of its biologic properties[J]. Chin J Cancer, 1987, 6: 235-238.

(2) Zhong X Y, Chen Y X, Ye S F. Establishment of two cell sublines from SWO-38 glioma cells: an immunohisto-chemical and ultrastructural Study[J]. Int J Modern Cancer Therapy, 2000, 3: 34-37.

(3) Zhu HL, Xie SM, Fang M, Zhang JJ, Weng ZP, Zhong XY. 4E-BP1 regulates the sensitivity of human glioma cells to chemotherapy through PI3K/Akt/mTOR-independent pathway. Neuropathology. 2014 Jun;34(3):227-35.

(4) Yan YR, Xie Q, Li F, Zhang Y, Ma JW, Xie SM, Li HY, Zhong XY. Epithelial-to-mesenchymal transition is involved in BCNU resistance in human glioma cells. Neuropathology. 2014 Apr;34(2):128-34.

(5) Xie SM, Fang M, Guo H, Zhong XY. Silencing of MGMT with small interference RNA reversed resistance in human BCUN-resistant glioma cell lines. Chin Med J (Engl). 2011 Sep;124(17):2605-10.

(6) Zhang Y, Zheng S, Ngai SM, Zheng W, Li J, Chen T, Zhong X. A novel selenadiazole derivative induces apoptosis in human glioma cells by dephosphorylation of AKT. Chem

Pharm Bull (Tokyo). 2014;62(10):994-9.

(7) Wang C, Fang M, Zhang M, Li W, Guan H, Sun Y, Xie S, Zhong X. The positive correlation between DJ-1 and β-catenin expression shows prognostic value for patients with glioma. Neuropathology. 2013 Dec;33(6):628-36.

(8) Wang MH, Lin CL, Zhang JJ, Weng ZP, Hu T, Xie Q, Zhong XY. Role of PTEN in cholera toxin-induced SWO 38 glioma cell differentiation. Mol Med Rep. 2013 Jun;7(6):1912-8.

(9) Lin C, Liang Y, Zhu H, Zhang J, Zhong X. R280T mutation of p53 gene promotes proliferation of human glioma cells through GSK-3β/PTEN pathway. Neurosci Lett. 2012 Oct 31;529(1):60-5.

(10) Xie YK, Huo SF, Zhang G, Zhang F, Lian ZP, Tang XL, Jin C. CDA-2 induces cell differentiation through suppressing Twist/SLUG signaling via miR-124 in glioma. J Neurooncol. 2012 Nov;110(2):179-86.

(11) Fang M, Zhong XY, Du B, Lin CL, Luo F, Tang LJ, Chen J. Role of DJ-1-induced PTEN down-regulation in migration and invasion of human glioma cells. Chin J Cancer. 2010 Dec;29(12):988-94.

(12) Lin CL, Wang MH, Qin YF, Fang M, Xie BB, Zhong XY. Differentiation of SWO-38 glioma cells induced by CDA-2 is mediated by peroxisome proliferator-activated receptor gamma. J Neurooncol. 2009 Oct;95(1):29-36.

(13) Wang MH, Zhong XY, Lin CL, Xie YK, Jia JP, Li SM, Mi C. [Expression of peroxisome proliferator-activated receptor gamma in glioma]. Nan Fang Yi Ke Da Xue Xue Bao. 2008 Mar;28(3):444-6. Chinese.

(14) Lin J, Yang JC, Tan L, Luo SQ. [Sensitization of human glioma SWO cell line to tumor necrosis factor-induced apoptosis by blocking phospholipase C-gamma1 signaling pathway]. Nan Fang Yi Ke Da Xue Xue Bao. 2006 Mar;26(3):266-9. Chinese.

(15) Zheng G, Zhang JR, Liu FQ, Wang XW, Zhang XY. [Molecular mechanism of antisense glycosyltransferase oligonucleotide inhibiting human gliomas cell line SWO-38]. Ai Zheng. 2002 Oct;21(10):1095-9. Chinese.

(16) Zheng G, Zhang JR, Liu FQ, Rao ZG, Wang XW, Zhang XY. [Effects of lipofectin-mediated alpha1,4Gal T antisense oligonucleotide on human glioma cell line SWO-38]. Di Yi Jun Yi Da Xue Xue Bao. 2002 Apr;22(4):299-302. Chinese.

Effect of Chlorpromazine on glioma cell line proliferation.

To examine whether other anti-depressant drugs might have similar effect with TFP, an anti-depressant drugs Chlorpromazine, one of the member of Phenothiazine, was used to examine whether has similar effect with TFP. Glioma cell line U251 was treated with Chlorpromazine (0 μM, 2 μM, 4 μM, 6 μM, 8 μM and 10 μM), and cell viability was measured 24 hours later. A low concentration of Chlorpromazine (2 μM and 4 μM) treatment resulted in a statistically significant increase in the viability of U251 (Fig. S3, $p < 0.05$).

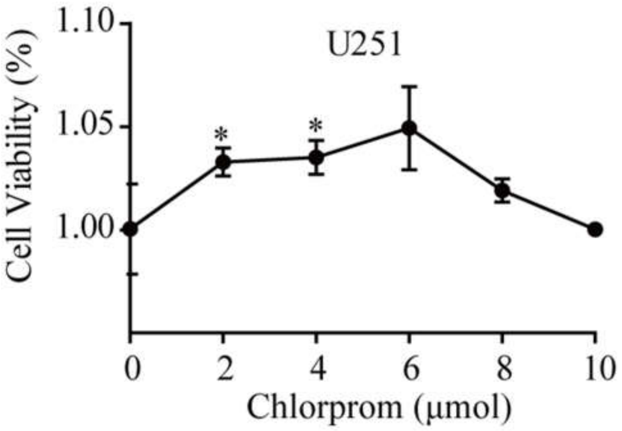

Fig. S3. The effect of different concentrations of Chlorpromazine (0 μM, 2 μM, 4 μM, 6 μM, 8 μM and 10 μM) on cell viability of glioma cell line U251. Data are the mean ± SD deviation of triplicate determinations.
